# Feasibility of Delivering a Dance Intervention for SubAcute Stroke in a Rehabilitation Hospital Setting

**DOI:** 10.3390/ijerph120303120

**Published:** 2015-03-16

**Authors:** Marika Demers, Patricia McKinley

**Affiliations:** 1 School of Physical and Occupational Therapy, McGill University, 3654 Promenade Sir-William-Osler, Montreal, QC H3G 1Y5, Canada; E-Mail: patricia.mckinley@mcgill.ca; 2 Feil and Oberfeld Research Center, Jewish Rehabilitation Hospital, Centre for Interdisciplinary Research in Rehabilitation of Greater Montreal, 3205 Place Alton-Goldbloom, Laval, QC H7V 1R2, Canada

**Keywords:** feasibility, dance intervention, rehabilitation, stroke, adjunct therapy

## Abstract

Dance can be a promising treatment intervention used in rehabilitation for individuals with disabilities to address physical, cognitive and psychological impairments. The aim of this pilot study was to determine the feasibility of a modified dance intervention as an adjunct therapy designed for people with subacute stroke, in a rehabilitation setting. Using a descriptive qualitative study design, a biweekly 45-min dance intervention was offered to individuals with a subacute stroke followed in a rehabilitation hospital, over 4 weeks. The dance intervention followed the structure of an usual dance class, but the exercises were modified and progressed to meet each individual’s needs. The dance intervention, delivered in a group format, was feasible in a rehabilitation setting. A 45-min dance class of moderate intensity was of appropriate duration and intensity for individuals with subacute stroke to avoid excessive fatigue and to deliver the appropriate level of challenge. The overall satisfaction of the participants towards the dance class, the availability of space and equipment, and the low level of risks contributed to the feasibility of a dance intervention designed for individuals in the subacute stage of post-stroke recovery.

## 1. Introduction

Stroke is a leading cause of disability worldwide [1]. In Canada, the prevalence of stroke is estimated to 50,000 strokes each year [2]. Stroke is a complex medical condition that can lead to physical, psychological and cognitive impairments impacting on activity and social participation [3]. Exercise interventions that improve mobility, balance, and quality of life are needed for individuals with residual movement impairments post-stroke [4]. To address the needs of individual post-stroke with multiple impairments, many novel rehabilitation interventions are emerging. One of the innovative interventions that has been used for older adults and individuals with neurological conditions is dance [5,6,7]. 

Recently, dance has shown to be a promising treatment modality, because of its pertinent and lasting effect on psycho-emotional perspectives that may greatly enhance its use as a physical activity in rehabilitation [8], in addition to providing cardiovascular benefits [9]. There is increasing evidence to support the use of dance in rehabilitation to address various impairments. A recent systematic review by Keogh *et al.* [10] suggested that the practice of dance can present multiple physical benefits for older adults (grade B level of evidence): aerobic power, muscle endurance, strength, and flexibility of the lower body; static and dynamic balance/agility; and gait speed. For people with neurological disorders, dance can bridge the gap between an enjoyable social activity and a therapeutic exercise dispensed at the proper intensity to target strengthening, mobility and balance [11]. It may be a particularly valuable intervention to help older adults stay healthy and connected with others [12]. In a recent study by Hackney *et al.* [7], 30 h of adapted tango lessons were offered to an individual in the chronic phase of stroke recovery, presenting with spastic hemiplegia in lower and upper extremities and age-related macular degeneration. After the intervention, improvements were noted on the Berg Balance Scale, 30-s chair stand Timed Up and Go (single, manual, and cognitive conditions), 6-min Walk Test and the Functional Reach Test. In a meta-analysis on the effectiveness of dance therapy, Ritter and Low [11] suggested that socialization, emotional expression, body-awareness, movement quality and coordination can be attributed to dance. Similar to numerous complex sensorimotor activities (e.g., sport, fitness and physical performance), dance requires the integration of spatial patterns, rhythm, synchronization to external stimuli and whole-body coordination [13]. While dancing, the focus of participants is on postural control, voluntary stepping strategies, whole-body coordination, and somatosensory awareness. Performing dance also demands a type of interpersonal coordination in space and time that is almost nonexistent in other social contexts [14]. 

The use of dance in post-stroke rehabilitation is consistent with the Canadian Best Practice Stroke Recommendations for Stroke Care [15] stating that patients should regularly participate in an aerobic exercise program that takes into consideration the functional limitations and impairments (evidence level B for early stroke). While there is evidence supporting the use of dance for older adults and individuals with disabilities, most of the dance interventions are delivered in the community or in laboratory-controlled settings, rather than in a hospital setting. There is a need to determine the extent to which a four-week dance intervention delivered for individuals post-stroke is feasible, in addition to the usual care, in the context of functional intensive hospital-based rehabilitation. More specifically, the feasibility of incorporating a dance intervention in a hospital-based rehabilitation setting was defined as: (1) participants’ tolerance to the type of exercise, the intensity and the frequency of the dance intervention; (2) level of risks (occurrence of adverse events); (3) participation; (4) participants’ satisfaction with the intervention; (5) availability of equipment and space, and support from the organization and the staff. A secondary objective was to identify the obstacles to implementing this intervention and ensuring its sustainability. Because this project was a pilot study with a small sample size, the focus of this paper is not a statistic comparison of the improvement in balance for participants or establishing if the improvements noted are explained by the dance intervention.

## 2. Experimental Section 

This study used a descriptive qualitative design. A biweekly dance intervention was conducted between February and July 2011 at the Jewish Rehabilitation Hospital (JRH). A modified 45-min dance class was delivered in a group format twice a week over 4 weeks to individuals in the subacute phase of post-stroke recovery. It was given in supplement to the usual care. The participants could join the group at any time. The JRH is a university-affiliated functional intensive rehabilitation centre offering general and specialized rehabilitation services to internal and ambulatory clientele. The care was organized by program (stroke, traumatic brain injuries, work injuries, *etc.*) and was client-centred. The interdisciplinary team consisted of nurses, physical and occupational therapists, social workers, speech-language pathologists, dieticians, and neuropsychologists (the team’s composition varied based on the patient’s needs). The usual care consisted of daily 45-min sessions of occupational and physical therapy. Based on the client’s need, speech-language pathology or psychology consultations were also offered (frequency determined by the rehabilitation goals). In addition, clients had the opportunity to participate in recreational activities and educational sessions about stroke and fall prevention. No formal aerobic exercise group was offered. In the stroke program, all interventions were delivered in an individual format, except for educational sessions. The JRH had 41 beds designated for the rehabilitation of the neurological clientele, including stroke and other neurological conditions. The overall goal of the dance intervention was to increase the intensity of treatment offered to the clientele post-stroke and to implement an aerobic exercise program, delivered in a group format, focusing on physical and cognitive impairments.

### 2.1. Participants 

All clients followed at the JRH for a subacute stroke (between 1 and 6 months post-stroke) met the inclusion criteria for this pilot study. Participants were included in the study regardless of their co-morbidities or the medication taken, as long as they were in a stable medical condition. All clients with severe motor apraxia, severe mixed aphasia, tetraplegia or who presented with a poor tolerance to a group setting or significant behavioural problems were excluded from this study. Clients who did not have the endurance to tolerate a minimum of two 45-min of treatment per day were also excluded as the participants still had to undergo their usual care in addition to this research project. To ensure a sufficient number of participants in each dance session, the group was open to individuals with other medical conditions with similar impairments, such as musculo-skeletal disorders, multiple sclerosis or traumatic brain injury. No data were collected on those participants because they did not consistently participate in each dance session and no formal objective was defined. 

### 2.2. Recruitment of Participants 

The ethics board of the Centre for Interdisciplinary Research in Rehabilitation of Greater Montreal approved this pilot project. Written informed consent was obtained from each participant. None of the participants received any form of compensation and they did not have to pay to receive the dance classes. If the family members were present at the time of the dance classes, they were encouraged to observe the dance class, assuming that the participants were in agreement. 

The overall objective, and the inclusion and exclusion criteria for this pilot project were presented to the interdisciplinary team working in the Stroke program at the JRH. The physical and occupational therapists were instructed to suggest participating in this research project, as an adjunct therapy, to the clients who met the inclusion criteria. The potential participants had the opportunity to observe a dance class prior to consenting to participate in this research project. 

### 2.3. Dance Intervention 

The dance style used for this group was a combination of jazz dance and merengue. Jazz dance was selected, because this style combines whole-body movements requiring flexibility, balance and endurance, with perceptive-cognitive skills. By using choreography or short routine, it allowed the repetition of the dance steps learned to foster memorization and added the additional challenge of remembering a sequence of steps. Jazz dance encompasses various other dance styles, such as swing and rock-and-roll, and it is commonly performed in ballrooms, making it easier for participants to relate to a previous dance experience. The basic steps of the merengue were also incorporated into the dance intervention, because the steps are simple, easy to learn and promote the transfer of weight from one side to the other. Dance exercises were targeting flexibility, balance, endurance, upper extremity function, perception (visual imagery and incorporation of the affected side for individuals with hemineglect) and memory. The Canadian Best Practice Recommendation for Stroke Care [15] was integrated in the development of the dance program. The dance steps demonstrated by the dance instructor were the same for all participants. However, given the great variability of each participant’s functional ability, the complexity and the intensity of the dance exercises were progressed according to each participant to achieve an appropriate challenge at moderate treatment intensity (measured with the Borg Rating of Perceived Exertion Scale). All the dance steps could be performed in sitting or standing position. The sessions were led by an occupational therapist (OT) that had previous dance experience in various dance styles. Depending on the number of participants, assistance to the dance instructor was provided by one or two OT students, typically using a ratio of three participants for one therapist. The OT students were already trained to analyse an activity to ensure patient safety, minimize the risk of fall and modify the exercises for each participant, when needed. The equipment required to run the dance class included chairs and a portable media player with music. A therapeutic plinth adjustable in height was also used for all participants who did not have enough balance to perform the activity in standing position, but could sit down without back support. The dance class was conducted in a small portion of the occupational therapy department (5 m^2^). To avoid interference with the usual care, the dance class was given at the end of the working day (from 3:15 to 4:00 p.m.). This time slot was the least busy of the day, which allowed minimized disturbance for other therapists and their patients in terms of space and noise from the music. The structure consisted of five components: warm up, technical exercises, improvisation, a short routine and a cool down. The structure of the dance class is described in detail in Table 1.

**Table 1:** Structure of the modified dance class designed for individuals post-stroke.

Main Sections of the Dance Intervention	Designated Time	Components of the Exercises
Warm-up exercises	10 min	Range of motion of all joints (from neck to toes) and slow passive stretching of the most affected upper extremity using the least affected upper extremity
		Neck, wrist and ankle rolling
		Shoulder elevation
		Basic dance step (e.g., step touch) to increase heart rate
Technical exercises (One exercise taught per session)	10 min	Trunk rotation
		Weight shifting
		Moving the hips (side-ways)
		Basic step of the meringue
		Step-touch side-ways
		Step-touch forward-backward
		Sliding to the side
		Twist
Directed improvisation	10 min	Participants were instructed to move freely according to some directions (large and big movements, bilateral movements only, *etc.*)
Dance routine	10 min	Routine of 30–45 sec, performed with the dance instructor acting as a model for the participants
		Basics skills linked together
		Integration of the dance step learned in the session
		Inclusion of the most affected side in the dance movements
Relaxation	5 min	Range of motion of all joints and breathing exercises

### 2.4. Modification to the Dance Class Specifically for Individuals Post-Stroke 

To target the dance intervention for individuals post-stroke, weight bearing on the most affected side and integration of the impaired limbs were strongly encouraged. Participants were encouraged to find solutions to allow them to perform a movement they found difficult due to physical limitations secondary to stroke. During the improvisation, participants were instructed to be creative in the movements performed to generate a larger repertoire of dance steps. Suggestions of alternative dance steps were also proposed to the participants, either to encourage them to use a larger range of movements or to adjust the level of challenge. For the participants with severe decreased balance, the dance steps were performed in sitting position. These participants were encouraged to move their arms and legs, move the wheelchair forward and backwards and perform flexion/extension or lateral flexion of the trunk. Participants with poor standing balance were paired with a dance instructor (or a family member) who held their hands to increase their support and provide tactile input for increasing stability [16]. External support was also offered and participants were encouraged to increase their base of support. Bilateral movements, passive mobilization of the affected extremity and weight bearing were encouraged for participants with severe hemiparesis. To facilitate the memorization of the dance steps for individuals with cognitive deficits, the routine was taught step-by step and each dance step was given a name or an image. When performing the routine, a dance instructor demonstrated the routine in front of the group and provided cues for the following movements. 

### 2.5. Outcome Measures 

The dance instructor kept a journal containing the feedback of the participants on the dance intervention. She also completed an unstandardized observation grid for each participant, containing the following elements: participation and social interaction (interaction with the other participants, ability to follow instructions), balance (use of external support, loss of balance), endurance (portion of the dance class performed in sitting/standing position, rest period needed), rhythm (ability to follow the music rhythm), quality of movements (use of the impaired limbs, fluidity and smoothness of movement, ability to reproduce the dance steps), and memorization of the routine (with or without model). Because participants continued their rehabilitation, which aimed, among others, at improving balance, it was expected that participants’ balance would improve over time. To document changes in balance over time, the treating physical therapist administered the Berg Balance Scale (BBS) in the week prior to and following the dance intervention, since it was routinely used at the JRH. The socio-demographic characteristics of the participants and performance in standardized evaluations were taken from their medical chart. Thus, different assessments were used to determine the presence of cognitive deficits from one participant to another, such as the Montreal Cognitive Assessment (MoCA), the Cognitive Assessment Scale for the Elderly (CASE), the Rivermead Behavioural Memory Test (RBMT) and the Cognitive Competency Test (CCT). The analysis of the OTs was used to qualify the cognitive deficits of each participant (light, moderate or severe cognitive impairments). 

## 3. Results

### 3.1. Participation 

All healthcare professionals working in the Stroke program expressed openness to suggest this research project to the clients who met the inclusion criteria. However, clinicians needed frequent reminders to suggest participating in this research project. Of the 41 rehabilitation beds designated to the neurological clientele, approximately 10 clients were eligible to participate in this study at any given time. Over a period of 20 weeks, 16 participants were recruited for the class and nine participants completed eight dance sessions. None of the participants had any formal training in dance prior to their stroke, defined as having taken more than one year of dance lessons in adulthood. Of the participants who dropped out from the dance classes, four participants were discharged from rehabilitation before the completion of eight dance sessions. Three other participants dropped out of the dance class after trying one or two sessions, because they did not like this intervention and were not engaged in the dance classes. The individual characteristics of the participants who completed the program are presented in Table 2. The mean age of those participants was 63.7 ± 11.7 years (range 47–78) and the majority of the participants were female. Participants were taking 5.9 ± 1.5 medications daily, excluding medication taken when required. 

**Table 2:** The characteristics of the participants who completed eight dance sessions (n = 9).

Age	Gender	Diagnosis	Months Post-Stroke	Hemisphere Lesion	Pre BBS Score ^1^	Post BBS Score	Cognitive Deficits	Assistive Device Used for Ambulation	Number of Medication Taken
66	Male	MCA ^**2**^ ischemic stroke	6	Left	48	48	Light	Simple cane	6
74	Female	MCA ischemic stroke	2	Right	11	28	Light-Moderate	Wheelchair	7
58	Female	MCA ischemic stroke	1	Left	45	47	Light	Simple cane	4
47	Male	Brain stem ischemic stroke	3	Left	26	42	Light-Moderate	Wheelchair and quadripod cane	4
62	Female	Ischemic stroke (affected brain region not specified)	2	Right	40	47	Light-Moderate	Quadripod cane	7
69	Female	Brain stem stroke	1	Right	5	50	Light	Wheelchair and quadripod cane	6
78	Female	Fronto-parietal ischemic stroke	1	Right	56	56	Moderate	No assistive device	8
73	Female	Lacunar stroke of the thalamus and the internal capsule	2	Left	5	40	Light	Wheelchair and quadripod cane	7
74	Female	MCA haemorrhagic stroke	4	Right	5	24	Light	Wheelchair	4

**^1^** BBS: Berg Balance Scale; **^2^** MCA: Middle Cerebral Artery.

The number of individuals who participated in the dance intervention varied from three to eight at one time. This number included the study participants, individuals with other medical conditions and former participants (n = 5) who continued to participate in the dance intervention after the completion of the program, as out-patients. For all participants with low BBS initial score (<40/56), the BBS improved over time, as they continued to receive their usual care in addition to receiving the dance intervention. 

### 3.2. Frequency, Duration and Intensity

For this study, nine participants were able to complete a 45-min dance class in addition to their usual care. A 45-min session was long enough to respect the structure of a usual dance class and allowed participants to learn a short routine with a few dance steps. Participants often experienced mild to moderate fatigue after 45 min of moderate intensity dance exercises. However, beyond this time, a significant increase in fatigue was reported, even if the exercises were modified to allow more resting time. In the context of functional intensive rehabilitation, the frequency of two sessions per week in addition to the usual treatment was feasible and realistic in terms of the availability of therapists and turnout to the dance class. The intensity of the treatment was not standardized but graded for each participant according to endurance levels to provide moderate treatment intensity. 

### 3.3. Space

The dance class can be performed in an open or closed room. For this study, the selection of an open room rather than a separate private area had advantages for the dance program, but also some inconvenience or the other OTs. One of the advantages for the participants was that they enjoyed performing the learned choreography for an audience composed of therapists, family members or other clients in the treatment room. Another advantage was the ability to stimulate the interest of other clients and possibly recruit new participants. Because of the proximity of the class to the other therapists, additional assistance was available if required. The inconvenience for the other therapists using the room included a temporary loss of space in the treatment room and the possible disturbance to their clients due to the music. Since the dance class was performed in a corner of the therapy department, this prevented from having anyone walk through the dance class while it was taking place and minimized disruption. No formal complaints were received from either the therapists or the patients for losing space or being disturbed by the music. 

### 3.4. Music Selection

The songs that solicited the most participation were the popular hits of the 50s’ to 80s’. Participants reported that they preferred songs with a fast pace and a strong beat, whatever the music style. Most of the time, the music was selected based on the participant’s preference in a predetermined playlist. 

### 3.5. Occurrence of Adverse Events

The main risks for participating were the risk of fall and an increase in fatigue. No negative consequences to participation were experienced during or after the dance classes, except increased fatigue: *“I worked hard, I’m tired”.* However, when surveyed, the participants did not feel more tired than after their usual therapies: *“I feel as tired as after a good session of physio”*. During the dance class, the participants did not want to stop even if they were becoming fatigued. 

### 3.6. Participants’ Perception

Participants reported that dance was a challenging, but enjoyable activity, and it was a great complement to their usual therapies. They also mentioned that they enjoyed interacting with the other participants. Two participants expressed that the dance intervention gave them confidence to move in their own body and dance in an informal social context. One participant said *“(The dance intervention) allowed me to meet other people with the same kind of problems as me”*. Another one expressed that *“The exercises are not easy, but I have a lot of fun to attend those classes”*. Participants also spontaneously reported an improvement in their standing balance and a decreased fear of falling: *“I feel safer to move when I'm standing”*, *“I can see that my balance is better, because of the dance group”*. All participants reported that they liked to perform in front of a small audience because they feel “proud of their accomplishment”. They stated that it was their favourite part of the dance class. Eight participants mentioned being satisfied with the dance intervention and one expressed being “neutral”. Of the seven participants who dropped from the study, three did not enjoy the intervention and consequently dropped the classes. The remaining four participants expressed being satisfied with the intervention, but received their discharge before the completion of eight dance classes.

### 3.7. Support from Staff and the Organization

The stroke team and the program coordinators were open to this new intervention, because it was perceived as a fun and innovative approach to increase treatment intensity, and the classes did not interfere with the usual care. When the intervention was implemented, the program coordinators and the research team worked closely to select an appropriate space for the dance classes. When the dance classes were implemented, the OT aid provided her support by helping the transportation of participants and the preparation of the room. The OTs were open to the dance classes, even if a portion of the occupational therapy treatment room was not available during the dance classes. The treating physical and occupational therapists collaborated with the dance instructor to progress the level of difficulty based on each client’s improvements and to target the individualized treatment objectives. Overall, positive feedback and encouragements were received from the staff. 

## 4. Discussion

The aim of this study was to determine the feasibility and application of a novel dance intervention to address physical and cognitive impairments in individuals post-stroke. Based on the results, an adjunct modified dance class was feasible in the context of functional intensive in-hospital rehabilitation for individuals in the subacute phase of post-stroke recovery. The frequency, duration and intensity of the dance classes were well tolerated by the participants, which is consistent with other studies using dance for individuals with neurological disorders [6,7,17,18,19] or exercise-based intervention in the subacute phase of stroke recovery [20,21,22]. The results suggested that dance could be performed in a limited space, contributing to its use in rehabilitation. Throughout the implementation of the dance classes and the duration of this pilot study, the program coordinators and the staff were open and supportive. However, as suggested in a study investigating the facilitators and barriers to implementing a dance intervention, all clinicians and program coordinators might not perceive the benefits of using dance in rehabilitation or adopt an open attitude [23], which can represent an important obstacle to the recruitment of participants and its sustainability. The level of risks associated with the dance intervention was low, as no adverse events occurred during or after the intervention. The potential risks of dance-based exercises should be anticipated and addressed to ensure participants safety [8]. Because dance is an enjoyable activity that is not always perceived by the participants as typical physical exercise, they can become fatigued. Therefore, the monitoring of the level of fatigue and exertion with a visual-analogue scale at different times during the class can prevent excessive fatigue. 

Concerning participation, a high attrition rate (43.8%) was noted. This attrition rate can be partially explained by the short length of stay in in-patient rehabilitation, as 4/7 participants were discharged before the completion of the program and were not able to pursue their participation as outpatients, despite the enjoyment of the dance classes. Thus it is suggested that patients should be enrolled in the dance class early in the rehabilitation process. Since the majority of the participants who completed the program continued to attend the dance classes after the completion of the study, the duration of the program could be different for each participant and determined based on the individualized treatment plan. As mentioned by Rabbia [24], dance can be intimidating for people who have not participated in dance classes in the past. This could have contributed to the lack of engagement of the three participants who dropped the class and could have influenced enrolment. The incorporation of this modality into the individualized treatment plan could help to strengthen the sense of engagement and could help in the gradation of the dance steps for each participant. One of the factors influencing participation was the time at which the class was scheduled. To avoid interference with the usual care and scheduled therapy, the dance class was offered at the end of day. However, this time slot was also a period when patients were more tired or were receiving visitors. Another time slot could, potentially, have resulted in a better participation. 

Overall, 75.0% of the participants enjoyed the dance intervention. The dance intervention promoted social interaction, as it was performed in a group format. The collaboration and camaraderie between participants, the sense of accomplishment and the perceived improvements contributed to the satisfaction towards the intervention. Due to the important diversity in the participants’ impairments, the results suggest that the class can be taught to people across a spectrum of impairments. The changes in the BBS indicated that individuals in the subacute phase of stroke recovery could improve quickly, which illustrates the importance to progress the level of difficulty based on the participants’ improvements. Since the objective of this study was not to determine the changes attributed to the dance intervention, it is not possible to conclude that the changes observed are due to the dance intervention. Following the end of this pilot study, the dance intervention continued to be offered to the clients post-stroke at the site.

### Study Limitations 

The sample size for this pilot study (n = 16) was small. The small sample size can be due to the method of recruitment used (referral from occupational and physical therapists) and the number of eligible participants. Other more effective recruitment strategies could have resulted in a higher participation. Furthermore, the percentage of participants who tried one or two sessions and did not continue to participate contributed to the high drop out rate of this intervention. As the participants’ characteristics were taken from their medical file, incongruencies were observed for the cognitive testing measure. In addition, because the original consent form stated that the information of the participants who dropped from the study would not be retained, data were not kept on the socio-demographic characteristics of those participants, therefore making impossible the comparison between the individuals who participated and those who dropped from the dance classes. This study was not designed with a control group to compare the effect of the dance class to an additional treatment because of its feasibility study status. Future studies should explore the benefits and outcomes of the dance intervention for individuals post-stroke. 

## 5. Conclusions

Dance is a promising treatment intervention that can be used as an innovative adjunct therapy to target multiple impairments in individuals in the subacute stage of post-stroke recovery, in a hospital setting. This pilot project supports the idea that dance is feasible to integrate to the usual care in a context of functional intensive rehabilitation, as little equipment and space is needed. The results suggest that a dance intervention can be sustained over time, due to the support from the organization and the staff. The dance exercises and the choreography can be adapted to the capacities of each participant with various impairments to provide an appropriate challenge. Moreover, the participants perceived dance as an enjoyable social and physical activity, which contributes to treatment adherence. 
